# Special Issue “Immune Ontogeny and Vaccination in Early Life: How the Non-Human Primate Model Can Help Expand the Current Knowledge in Pediatric Immunology and Infectious Diseases Research”

**DOI:** 10.3390/vaccines9091014

**Published:** 2021-09-12

**Authors:** Nabila Seddiki, Roger Le Grand

**Affiliations:** Center for Immunology of Viral, Auto-immune, Hematological and Bacterial Diseases, UMR1184, IDMIT Department-CEA, Université Paris Saclay, 92265 Fontenay-aux-Roses, France; roger.legrand@cea.fr

The development of the immune system requires a number of changes that occur during the first months of life. The neonatal immune system is exposed to a large number of previously unseen antigens [1]. Newborn children are susceptible to infections because of the immaturity of their immune system, and there is now clear evidence that several factors, such as microbial exposure, antibiotic treatment, mode of delivery and breastfeeding, are involved in shaping immunity. However, we still need to understand the basis of this, and consequently be able to protect young children by developing better treatments and efficacious vaccines tailored to their immune system. A large number of studies have explored these avenues. However, the field still lacks a good animal model to obtain insights into immune ontogeny in early life and the development of immune-mediated diseases later in life. Due to the phylogenetic proximity and the high level of homology in the molecular structures, non-human primates (NHP) have a similar immune system organization to humans [2]. This preclinical model is indeed suitable to study and explore immune development early in life and extrapolate the findings to human immune systems in the most comprehensive way, encompassing not only its impact on emerging and re-emerging infectious diseases and vaccinology, but also its close interaction with microbiota. We are currently establishing a program for pediatric infections and immunology studies (PIP; Figure 1 below) at the Infectious Disease Models for Innovative Therapies (IDMIT) Department at the CEA, France, where the NHP model is central to the development of comprehensive studies that will explore immune ontogeny, immune pathogenesis, microbiomes, vaccination, and the transfer of maternal immunity. In this context, following a brainstorming meeting that gathered experts in the field of pediatric immunology and infectious diseases (held at the IDMIT in February 2020), we aimed to mark this event by editing a Special Issue in the *Vaccines* journal, entitled “Immune ontogeny and vaccination in early life: How the NHP model can help expand the current knowledge in pediatric immunology and infectious diseases research”. The goal of this Special Issue is to compile a series of reviews covering new progress in understanding the development and maturation of the immune system in early life, and how it will react and respond to infections and vaccines. Some of the participants also commented on the relevance of the NHP preclinical model in this context.

In this Special Issue, Tokuhara and Hikita [3] point to the possibility of using a cord blood-based approach to screen candidate vaccine adjuvants, as it is non-invasive and cost-effective, and argue that it reflects the neonatal innate immune system. They suggest that this system may become key to developing safe and effective vaccines for children. Sartoretti and Eberhardt discuss the importance of in vivo research and highlight the importance of using the NHP model, given its genetic proximity to humans as compared to other animal models, to better comprehend early-life immunity and maternal antibody transfer [4]. Indeed, in the field of HIV-1 infection, antibodies with different effector functions being transmitted from mother to child and their role in the pathogenesis of infected children is still unresolved. Dispinseri et al. studied the kinetics of neutralizing antibody (Nabs) responses in HIV-1-infected children (transplacentally acquired) in both slow and rapid progressors, following them from just after birth to five years on. They showed that persistent Nabs triggering viral escape and an increase in breadth and potency are exclusive to HIV-1-infected, slowly progressing children [5].

The use of the NHP model has been further emphasized by Joma et al. [6] to better decipher vertical transmission and the consequences of viral infections and massive inflammation on newborn neuro-development. This preclinical model is indeed ideal for long-term follow-up.

It is now well established that interactions between the immune system and the microbiome play a crucial role in human health. These interactions start in the prenatal period and are critical for the maturation of the immune system in newborns and infants. Nunez et al. [7] focus on the ontogeny of the immune system and its association with microbial colonization, and they underline the remaining questions that need to be addressed and how the NHP model could help to uncover this path.

The path to developing new pediatric vaccines against pertussis (whooping cough) has been discussed in two separate reviews by Saso et al. (Saso et al., Vaccine-Induced Cellular Immunity against *Bordetella pertussis:* 2 Harnessing Lessons from Animal and Human Studies to 3 Improve Design and Testing of Novel Pertussis Vaccines) and Locht [8,9]. The development of novel vaccines against pertussis is a challenge, and most vaccine candidates have not yet progressed further than testing in murine models. To effectively control pertussis, novel vaccines that protect against the disease and prevent *B. pertussis* infection and transmission are needed. Only a few studies have investigated the effect of some novel vaccines against nasal colonization, and only two of them have been tested in NHPs.

Our understanding of the immature immune system response facing an infection is still limited, and the current COVID-19 pandemic, which has shown that children are less severely affected than adults, remains unclear. The mechanisms involved remain incompletely understood but could include the rapid development of a robust innate immune response. In this Special Issue, Blanchard-Rohner et al. (Blanchard-Rohner et al., Pediatric COVID-19: clinical presentation, pathogenesis and prevention) discussed pediatric COVID-19 and the importance of vaccination as a strategy for global health [10]. The lessons learned and experience gained from the COVID-19 pandemic will be very important for the prevention and care of other infections affecting children, and for the pathogens that will cause future pandemics [10]. There is still, undoubtedly, a need to obtain insight into early-life immunity and the mechanisms governing it in physiology and infectious conditions. The NHP preclinical model is valuable when addressing these unanswered questions.

## Figures and Tables

**Figure 1:**
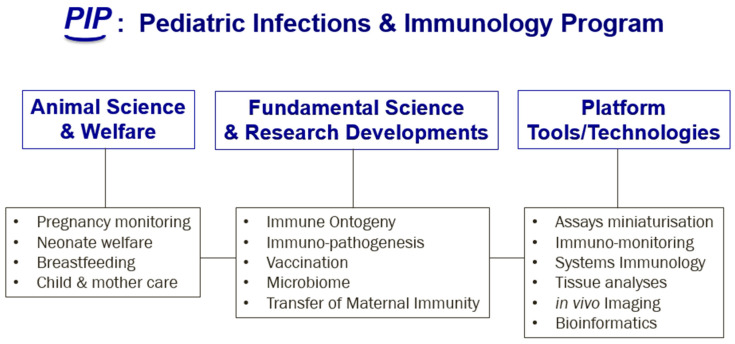
The Pediactric Infections and Immunology Program (PIP) is developped at IDMIT. Three main structures, namely “Animal Science and Welfare”, “Fundamental Science and Research Developments” and “Platforms, Tools and Technologies” were put in place to better decipher the development and maturation of the immune system in early life, and understand how it will react and respond to infections and vaccines. The non-human Primate (NHP) model is a valuable asset for translational research.

## References

[1] Olin A., Henckel E., Chen Y., Lakshmikanth T., Pou C., Mikes J., Gustafsson A., Bernhardsson A.K., Zhang C., Bohlin K. (2018). Stereotypic Immune System Development in Newborn Children. Cell.

[2] Lemaitre J., Naninck T., Delache B., Creppy J., Huber P., Holzapfel M., Bouillier C., Contreras V., Martinon F., Kahlaoui N. (2021). Non-human primate models of human respiratory infections. Mol. Immunol..

[3] Tokuhara D., Hikita N. (2021). Cord Blood-Based Approach to Assess Candidate Vaccine Adjuvants Designed for Neonates and Infants. Vaccines.

[4] Sartoretti J., Eberhardt C.S. (2021). The Potential Role of Nonhuman Primate Models to Better Comprehend Early Life Immunity and Maternal Antibody Transfer. Vaccines.

[5] Dispinseri S., Cavarelli M., Tolazzi M., Plebani A.M., Jansson M., Scarlatti G. (2021). Continuous HIV-1 Escape from Autologous Neutralization and Development of Cross-Reactive Antibody Responses Characterizes Slow Disease Progression of Children. Vaccines.

[6] Joma M., Fovet C.M., Seddiki N., Gressens P., Laforge M. (2021). COVID-19 and Pregnancy: Vertical Transmission and Inflammation Impact on Newborns. Vaccines.

[7] Nunez N., Reot L., Menu E. (2021). Neonatal Immune System Ontogeny: The Role of Maternal Microbiota and Associated Factors. How Might the Non-Human Primate Model Enlighten the Path?. Vaccines.

[8] Saso A., Kampmann B., Roetynck S. (2021). Vaccine-Induced Cellular Immunity against Bordetella pertussis: Harnessing Lessons from Animal and Human Studies to Improve Design and Testing of Novel Pertussis. Vaccines.

[9] Locht C. (2021). The Path to New Pediatric Vaccines against Pertussis. Vaccines.

[10] Blanchard-Rohner G., Didierlaurent A., Tilmanne A., Smeesters P., Marchant A. (2021). Pediatric COVID-19: Immunopathogenesis, Transmission and Prevention. Vaccines.

